# Who would avoid severe adverse events from nasointestinal tube in small bowel obstruction? A matched case–control study

**DOI:** 10.1186/s12876-022-02405-8

**Published:** 2022-07-07

**Authors:** Hui Wang, Jun-rong Zhang, Shuai Chen, Ping Hou, Qing-feng Chen, Zong-qi Weng, Xin-chang Shang-guan, Bing-qiang Lin, Xian-qiang Chen

**Affiliations:** 1 Department of General Surgery (Emergency Surgery), Fujian Medical University Union Hospital, No.29 Xinquan Road, Fuzhou, 350001 Fujian Province China; 2 Immunotherapy Institute, Fujian Medical University, No.1 Xuefu bei Road, Fuzhou, 350122 Fujian Province China

**Keywords:** Nasointestinal tube, Small bowel obstruction, Indication, Severe adverse events, Risk score system

## Abstract

**Background:**

Nasointestinal tubes (NITs) have been increasingly used in patients with small bowel obstruction (SBO); However, severe adverse events (SAEs) of NITs might threaten the lives of patients. The indications of NITs need to be identified. This study was designed to explore the indications for the insertion of NITs in patients with SBO and to suggest the optimal strategies for individuals based on the outcomes of SAEs.

**Methods:**

After propensity score matching, 68 pairs were included (Success group and failure group). The occurrence of SAEs and the clinical parameters were compared between the SAE group and the non-SAE group. Independent risk factors were evaluated among the subgroups. A novel scoring system was established to detect the subgroups that would benefit from NITs insertion.

**Results:**

Successful implementation of NITs could avoid hypochloremia (*p* = 0.010), SAEs (*p* = 0.001), pneumonia (*p* = 0.006). SAEs occurred in 13 of 136 (9.6%) patients who accepted NITs insertion treatment. Risk factors for SAEs included tumors (*p* = 0.002), reduced BMI (*p* = 0.048), reduced hemoglobin (*p* = 0.001), abnormal activated partial thromboplastin time (*p* = 0.015) and elevated white blood cells (*p* = 0.002). A novel risk scoring system consists of hemoglobin before NITs insertion (95% CI 0.685, 0.893) and bowel obstruction symptoms relieved after NITs insertion (95% CI 0.575, 0.900) had the highest area under curve for predicting the occurrence of SAEs. We divided the risk score system into 3 grades, with the increasing grades, the rates of SAEs surged from 1.3% (1/74) to (6/11) 54.5%.

**Conclusion:**

NITs successfully insertion could avoid SAEs occurrence in SBO conservative treatment. SBO patients without anemia and could be relieved after NITs insertion could be the potential benefit group for this therapy.

**Supplementary Information:**

The online version contains supplementary material available at 10.1186/s12876-022-02405-8.

## Introduction

Small bowel obstruction (SBO), defined as a partial or complete blockage of the small intestine, is a common surgical emergency, accounting for 20% of the emergency surgical procedures of patients presenting with abdominal pain and approximately 300,000 hospitalizations in the United States annually [1, 2]. SBO causes high morbidity with an in-hospital mortality rate of 3% per episode and an average hospital stay of 8 days [3]. SBO usually resolves with conservative treatment but sometimes requires surgery if there is complete bowel obstruction, bowel perforation, severe ischemia, or condition deterioration during medical therapy. However, the indications for conservative treatment haven’t been defined and the SAE of failure by conservative treatment often puzzle clinicians. How to identify the conservative failure group in the early stage of disease is related to the short-term prognosis of patients.

Even though 50% of patients with SBO can be relieved with conservative treatment, such as intravenous hydration, decompression with a nasogastric tube or nasointestinal tube (NIT), and temporary elimination of oral nourishment [4–7], the diagnosis of delayed bowel strangulation during conservation is difficult. In addition, recurrences of adhesive small bowel obstruction after surgery can seriously dampen life quality for patients and create a dilemma for surgeons. Moreover, as the incidence of laparotomy management increases, the associated morbidities and postoperative complications, such as incisional infection, pneumonia, and intestinal leakage, have also increased [8, 9].

Wangensteen and Paine made early attempts to treat SBO with NITs in 1933 [10]. In 1938, Abbott and Johnston [11] reported a nonoperative technique of advancing a tube through the pylorus proximal to the obstruction, which showed an 80% success rate overall. Several studies have verified the effectiveness of NITs in treating SBO [6, 12–15], which leads to a reduction in intestinal pressure in the early stage, avoidance of intestinal ischemia, and transformation from acute SBO to subacute SBO. Nevertheless, electrolyte disturbance, intussusception and even intestinal perforation specifically caused by the insertion of NITs should not be ignored. However, the indications for the NITs insertion of patients with SBO have not been established.

In this study, patients who were treated with NITs decompression were retrospectively investigated to determine the independent risk factors for severe adverse events (SAEs) and to predict the patients who could benefit from decompression with NITs.

## Materials and methods

### Patient population

All patients (n = 210) who underwent ‘transnasal minimally invasive nasointestinal tube insertion’ at Fujian Medical University Union Hospital from January 2014 to November 2019 were recruited in this study. The diagnostic criteria for intestinal obstruction included abdominal pain, abdominal distention, vomiting, constipation, and a distended small bowel with air-fluid mixture images visible on computed tomography (Detailed data are presented in Additional file 1: Supplementary Figure). The study protocol was approved by the Institutional Review Board of our hospital, and all patients provided written informed consent for the procedure.

### Classification criteria

**Fig. 1:**
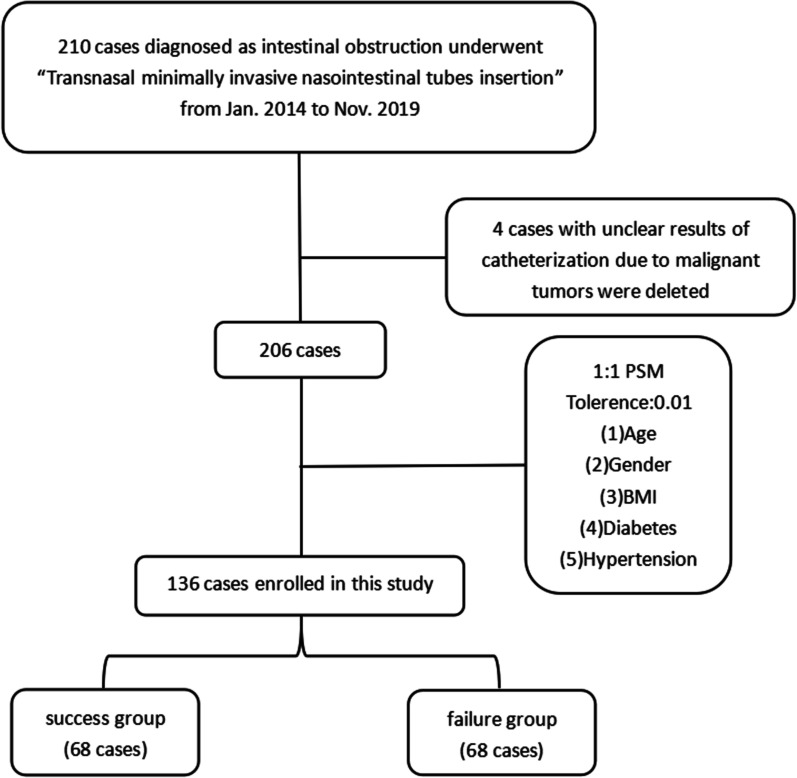
The workflow of this study

After applying strict selection criteria, 4 patients with unclear results of NITs insertion due to malignant tumors were excluded. Of these 206 patients, 121 patients were cured after NITs insertion, but the other 85 patients still needed a surgical intervention. Detailed information about our classification criteria is shown in Fig. 1.

### Instrument and procedures

The NIT used in our institution is a hydrophilic ileus tube (CLINY double balloon type; Create Medic Co., Ltd., Tokyo, Japan). The NIT is 300 cm in length and 16 Fr with three channels (suction channel, injection channel and balloon channel) and two balloons (anterior balloon and posterior balloon). The guidewire is 350 cm long and 1.24 mm in diameter [16]. The NIT guidewire was used to prevent the tube from twisting, and the tube was inserted into the jejunum for a minimum of 20 cm by following the stiff guidewire. After the guidewire is pulled out, 15 ml of sterile water is injected into the anterior balloon, and continuous bowel decompression is initiated with a negative pressure bag. The tube is propelled by bowel peristalsis until decompression treatment is completed [17].

### Definition of variants

The successful group was defined as the symptoms, such as abdominal pain, abdominal distension, and blockage of anal exhaust and defecation, relieved after NITs treatment. Meanwhile, the patients were able to tolerate semifluid diet and were discharged successfully. The failure group was defined as those for whom the patients’ symptoms could not be relieved after NITs insertion and required surgical intervention. Abdominal pain or distension relief was defined as the relief of the patient's subjective feelings within 3 days after NITs insertion. A novel scoring system was established: patients with a hemoglobin level lower than 110 g/L before NITs insertion were recorded as 1, patients with a hemoglobin level higher than 110 g/L were recorded as 0, patients whose symptoms could not be relieved by NITs insertion were recorded as 1, and those with relief of symptoms were recorded as 0.

The neutrophil, lymphocyte, monocyte, and platelet count from the peripheral blood tests and the inflammation indexes dependent on these factors were determined before and after NITs insertion. The NLR was calculated as neutrophil count/lymphocyte count [18].

Complications were subdivided into five grades according to the Clavien-Dindo classification system [19, 20]. Grade I was defined as complications not requiring additional interventions or only minor interventions such as vomiting; grade II was defined as complications requiring pharmacologic or other treatments, such as blood transfusion and total parenteral nutrition; grade III was defined as complications requiring surgical intervention or other interventional treatments; grade IV was defined as life-threatening complications, including central nervous system, cardiac, and pulmonary complications, renal failure, and those requiring intensive care unit (ICU) management; grade V was defined as death. The Clavien–Dindo grade I to grade III was classified as non-SAE, and Clavien–Dindo grade IV to grade V was classified as SAE.

### Statistical analysis

Between-group differences in qualitative variables were compared using the chi-squared test or Fisher’s exact test, and quantitative variables were compared using t-tests. Univariate and multivariate analyses were determined by logistic regression analysis. Based on the results of the multivariate analysis, HB and abdominal pain or distension relief were assigned as 0 or 1, and the ROC curve was drawn. All P values less than 0.05 were considered statistically significant. All statistical analyses and graphs were generated using SPSS 25.0 software.

## Results

### Baseline characteristics of the patients

**Fig. 2:**
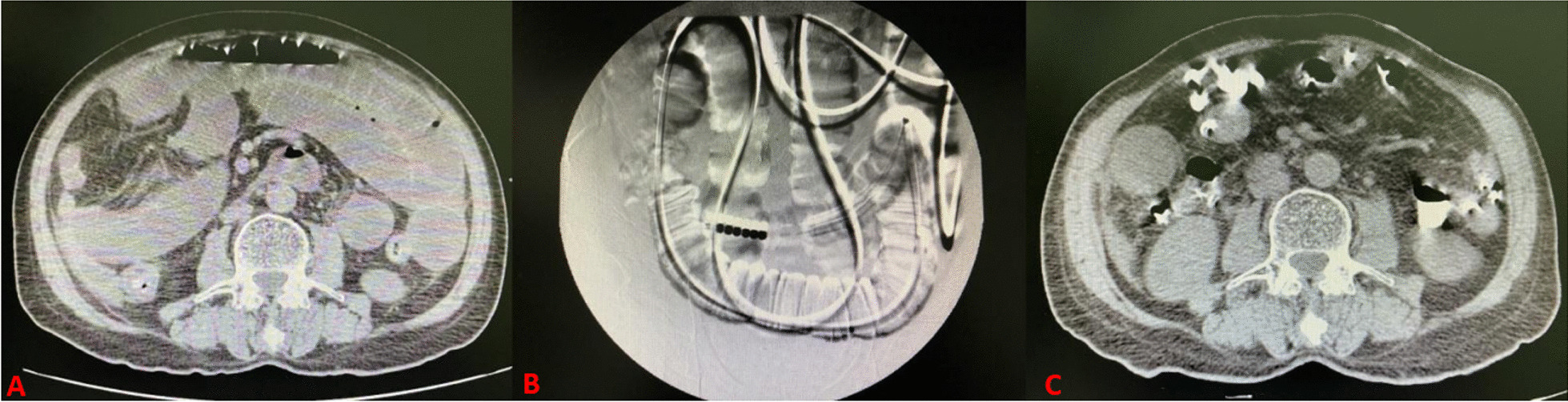
CT image of a patient with adhesive ileus with successful decompression after NIT insertion. **A** Before NIT insertion; **B** X—ray of completion of NIT insertion; **C** Three days after NIT insertion

**Fig. 3:**
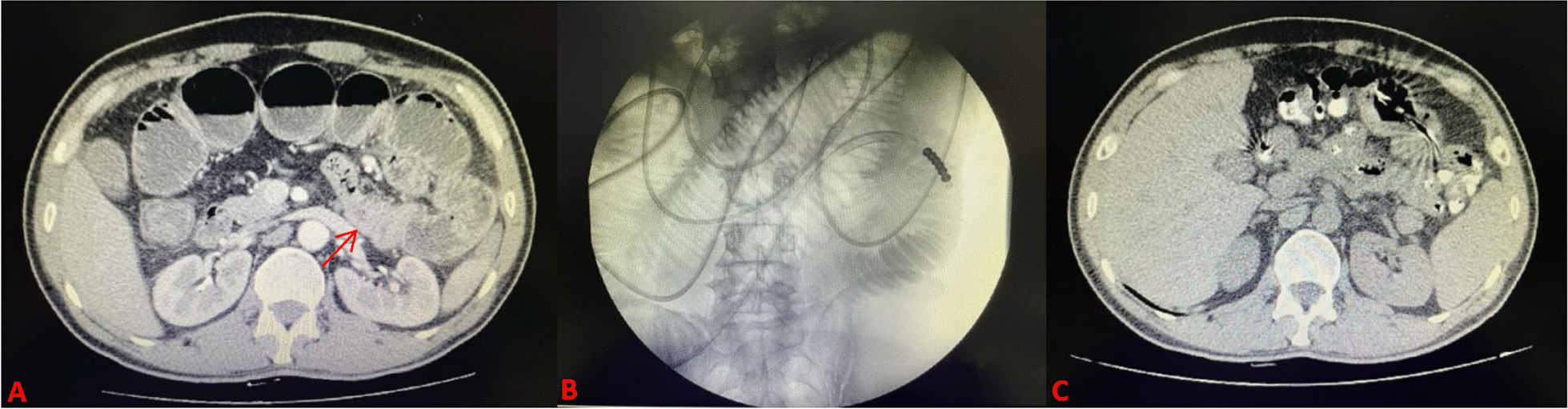
CT image of a patient with malignancy ileus who failed to decompress after NIT insertion. **A** Before NIT insertion(at the arrow is a tumor); **B** X—ray of completion of NIT insertion; **C** Three days after NIT insertion

Before PSM, age (*p* = 0.043), BMI (*p* = 0.137) and comorbidities such as diabetes (*p* = 0.175) were incomparable between the success and failure groups. After PSM, the clinical parameters including age, sex, BMI, tumor, electrolytes and inflammatory level, were precisely compared between the two groups (Table 1). Nontumor small intestine obstruction was more prone to success with NITs insertion (*p* = 0.015) (Figs. 2 and 3), and the bowel obstructive symptoms could be relieved, which indicated a high probability of success of NITs insertion (*p* < 0.001). Successful implementation of NITs could avoid hypochloremia (*p* = 0.010), SAEs (*p* = 0.001), pneumonia (*p* = 0.006). NITs insertion could shorten the hospitalization duration (*p* < 0.001) and fees (*p* < 0.001). With an increase in inflammatory biomarkers, NITs decompression is prone to failure.

**Table 1:** Clinical characteristics of patients

	Before propensity matching			After propensity matching		
Characteristics	Success group (n = 121)	Failure group (n = 85)	*p*	Success group (n = 68)	Failure group (n = 68)	*p*
Age, n (%)			0.043			0.864
< 60y	51 (42.1)	48 (56.5)		34 (50.0)	33 (48.5)	
≥ 60y	70 (57.9)	37 (43.5)		34 (50.0)	35 (51.5)	
Gender, n (%)			0.956			1.000
Female	38 (31.4)	27 (31.8)		19 (27.9)	19 (27.9)	
Male	83 (68.6)	58 (68.2)		49 (72.1)	49 (72.1)	
BMI, n (%)			0.137			0.970
18.5–23.9	100(82.6)	66(77.6)		58(85.3)	57(83.8)	
> 23.9	10 (8.3)	4 (4.7)		1 (1.5)	1 (1.5)	
< 18.5	11 (9.1)	15 (17.6)		9 (13.5)	10 (14.7)	
Diabetes, n (%)			0.175			1.000
None	100 (82.6)	76 (89.4)		60 (88.2)	60 (88.2)	
Yes	21 (17.4)	9 (10.6)		8 (11.8)	8 (11.8)	
Hypertension, n (%)	0.579			1.000		
None	111 (91.7)	79 (92.9)		62 (91.2)	62 (91.2)	
Yes	10 (8.3)	6 (7.1)		6 (8.8)	6 (8.8)	
Tumor, n (%)			< 0.001			0.015
None	107 (88.4)	57 (67.1)		58 (85.3)	46 (67.6)	
Yes	14 (11.6)	28 (32.9)		10 (14.7)	22 (32.4)	
Remission, n (%)	< 0.001			< **0.001**		
None	0 (0.0)	31 (36.5)		0 (0.0)	25 (36.8)	
Yes	121 (100.0)	54 (63.5)		68 (100.0)	43 (63.2)	
Cl^−^ level, n (%)			0.005*			0.010*
Normol (96–112)	102 (90.3)	57 (73.1)		56 (88.9)	42 (68.9)	
High (> 112)	1 (0.9)	3 (3.8)		0 (0.0)	3 (4.9)	
Low (< 96)	10 (8.8)	18 (23.1)		7 (11.1)	16 (26.2)	
CD, n (%)			< 0.001*			< 0.001*
1	119 (98.3)	50 (58.8)		67 (98.5)	40 (58.8)	
2	1 (0.8)	19 (22.4)		0 (0.0)	16 (23.5)	
3	1 (0.8)	2 (2.4)		1 (1.5)	1 (1.5)	
4	0 (0.0)	13 (15.3)		0 (0.0)	11 (16.2)	
5	0 (0.0)	0 (0.0)		0 (0.0)	0 (0.0)	
SAE, n (%)			< 0.001			**0.001**
0	119 (98.3)	69 (81.2)		67 (98.5)	56 (82.4)	
1	2 (1.7)	16 (18.8)		1 (1.5)	12 (17.6)	
Pneumonia, n (%)	0.001*			0.006*		
None	119 (98.3)	74 (87.1)		68 (100.0)	60 (88.2)	
Yes	2 (1.7)	11 (12.9)		0 (0)	8 (11.8)	
Fees (¥)	29,129	65,541	0.002	26,991	67,030	** < 0.001**
Length (days)	16 (± 12)	26 (± 14)	0.013	15 (± 13)	25 (± 14)	** < 0.001**
BMI, (kg/m^2^)	20.31 (± 2.16)	20.94 (± 2.03)	0.035	20.22 (± 1.92)	20.43 (± 1.82)	0.518
NLR, (ratio)	7.97 (± 8.75)	12.68 (± 20.13)	0.046	7.47 (± 7.58)	13.05 (± 21.97)	0.052*
WBC, (10^9^/L)	7.65 (± 4.25)	9.61 (± 7.32)	0.030*	7.58 (± 3.45)	9.13 (± 5.98)	0.071
Ne, (10^9^/L)	5.92 (± 4.12)	7.90 (± 7.14)	0.024*	5.77 (± 3.28)	7.44 (± 5.95)	**0.049**

Bold values indicate statistically significant

*CD* Clavien–Dindo grading of surgical complication, *SAE* severe adverse event, Remission, abdominal pain or distension relieved after NITs insertion; Cl^−^ level, level of chloride ion; Length, hospitalization duration; NLR, neutrophils/lymphocytes after NITs insertion; WBC, white blood cells hemoglobin after NITs insertion; Ne, neutrophils after NITs insertion; PSM tolerance: 0.02

All *p* values < 0.05 were considered statistically significant

*Fisher’s precise test

### SAE and non-SAE groups

According to the Clavien–Dindo grading of surgical complications (CD), SAEs occurred in 13 of 136 (9.6%) patients overall, including 4 (2.9%) with a poor general condition, 2 (1.4%) with a complicated condition and gave up treatment, 2 (1.4%) with respiratory failure, 1 (0.7%) with gastrointestinal perforation, 1 (0.7%) with severe pneumonia, 1 (0.7%) with anastomotic fistula, 1 (0.7%) with septic shock, and 1 (0.7%) with severe metabolic acidosis (Detailed data are presented in Additional file 2: Supplementary Table). There were 136 patients after PSM that were divided into the SAE and non-SAE groups (Table 2). Patients with tumors (*p* = 0.002) that could not be relieved after NITs insertion (*p* < 0.001) were more likely to develop SAEs. Patients with poor nutritional status (*p* = 0.048), low red blood cells (*p* = 0.032) and low hemoglobin (*p* = 0.001) were more likely to develop SAEs after NITs insertion. In addition, high inflammatory markers before insertion, such as white blood cells (*p* = 0.002), indicated that the patients were susceptible to SAEs.

**Table 2:** Influencing factors of SAE

	Non-SAE group(CD1-3) (n = 123)	SAE group (CD4-5) (n = 13)	*p*
Tumor, n (%)			0.002*
None	99 (80.5)	5 (38.5)	
Yes	24 (19.5)	8 (61.5)	
Remission, n (%)			< 0.001*
None	17 (13.8)	8 (61.5)	
Yes	106 (86.2)	5 (38.5)	
Na^+^ level, n (%)			0.075*
Normol (135–148)	86 (75.4)	5(38.5)	
Low(< 135)	26 (22.8)	4( 40.0)	
High(> 148)	2 (1.8)	1 (10.0)	
CD, n (%)			< 0.001*
1	107 (87.0)	0 (0.0)	
2	16 (13.0)	0 (0.0)	
3	0 (0.0)	2 (15.4)	
4	0 (0.0)	11 (84.6)	
5	0 (0.0)	0 (0.0)	
BMI, (kg/m^2^)	20.43 (± 1.80)	19.36 (± 2.25)	**0.048**
adRBC, (10^12^/L)	4.02 (± 0.96)	3.42 (± 0.72)	**0.032**
adHB, (g/L)	120.99 (± 22.34)	98.23 (16.87)	**0.001**
adAPTT, (s)	36.86 (± 6.46)	41.65 (± 5.17)	**0.015**
WBC, (10^9^/L)	7.95 (± 4.21)	12.29 (± 8.75)	**0.002**
Ne, (10^9^/L)	6.17 (± 4.09)	10.83 (± 8.71)	0.080*
RBC, (10^12^/L)	3.81 (± 0.83)	3.31 (± 0.71)	**0.039**
HB, (g/L)	115.06 (± 18.58)	96.77 (± 19.53)	**0.001**

Bold values indicate statistically significant

CD, Clavien–Dindo grading of surgical complications; Remission, abdominal pain or distension relieved after NITs insertion; Na^+^ level, level of sodium ion; adRBC, red blood cell before NITs insertion; adHB, hemoglobin before NITs insertion; adapt, activated partial thromboplastin time before NITs insertion; WBC, white blood cell after NITs insertion; Ne, neutrophils after NITs insertion; RBC, red blood cell after NITs insertion; HB, hemoglobin after NITs insertion

All p values < 0.05 were considered statistically significant

*Fisher’s precise test

### Univariate and multivariate analysis

The univariate analysis showed that tumor (*p* = 0.002), abdominal pain or distension relieved after NITs insertion (*p* < 0.001), red blood cells (*p* = 0.041), hemoglobin (*p* = 0.001), and activated partial thromboplastin time (*p* = 0.021) before NITs insertion were the independent risk factors for the occurrence of SAEs. Hemoglobin (*p* = 0.003) and white blood cells (*p* = 0.016) after NITs insertion were independent risk factors for the occurrence of SAEs.

The multivariate analysis showed that relief of bowel obstruction symptoms (*p* = 0.004) after NITs insertion and hemoglobin before NITs insertion (*p* = 0.014) independently affected the incidence rates of SAE in all patients (Table 3).

**Table 3:** Univariate and multivariate Cox regression analysis of risk factors for SAE

Characteristic	Univariate		Multivariate	
	OR (95%CI)	*p*-value	OR (95%CI)	*p*-value
Remission	0.100 (0.029,0.343)	** < 0.001**	0.123 (0.030,0.504)	**0.004**
Tumor	6.600 (1.982,21.980)	**0.002**		
adRBC, (10^12^/L)	0.599 (0.365,0.980)	**0.041**		
adHB, (g/L)	0.950 (0.921,0.9980)	**0.001**	0.958 (0.926,0.991)	**0.014**
adPLT, (10^9^/L)	0.997 (0.991,1.003)	0.362		
adAPTT, (s)	1.102 (1.015,1.197)	**0.021**		
HB, (g/L)	0.952 (0.922,0.983)	**0.003**		
RBC, (10^12^/L)	0.594 (0.350,1.006)	0.053		
WBC, (10^9^/L)	1.124 (1.022,1.237)	**0.016**		

Bold values indicate statistically significant

Remission, abdominal pain or distension relieved after NITs insertion; adRBC, red blood cell before NITs insertion; adHB, hemoglobin before NITs insertion; adPLT, blood platelet before NITs insertion; adapt, activated partial thromboplastin time before NITs insertion; HB, hemoglobin after NITs insertion; RBC, red blood cell after NITs insertion; WBC: white blood cell after NITs insertion

All *p* values < 0.05 were considered statistically significant

### The ROC curve and nomogram of risk factors

**Fig. 4:**
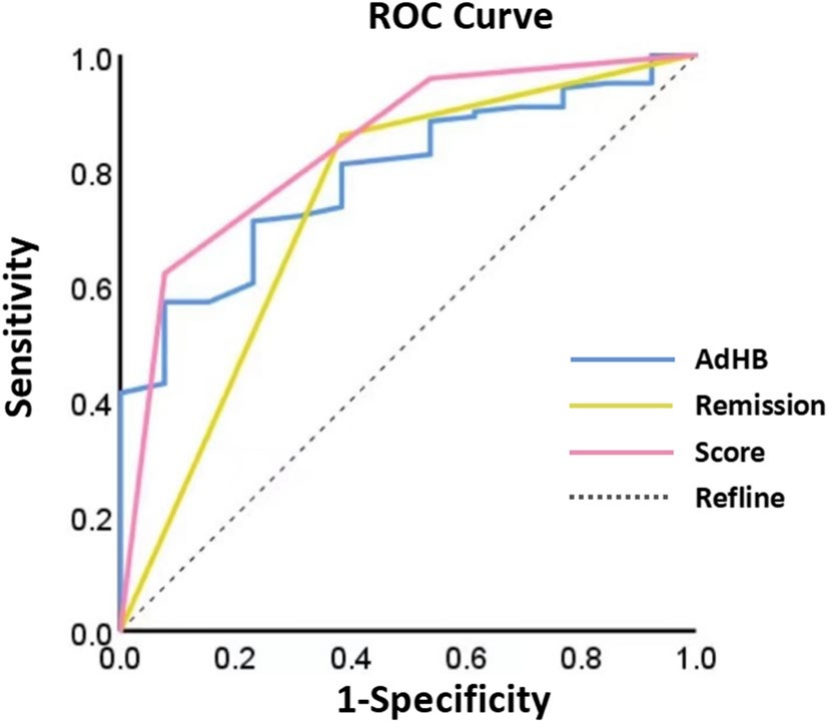
The ROC curve of abdominal pain or distension being relieved after NIT insertion, low hemoglobin before NIT insertion and ‘score’

**Fig. 5:**
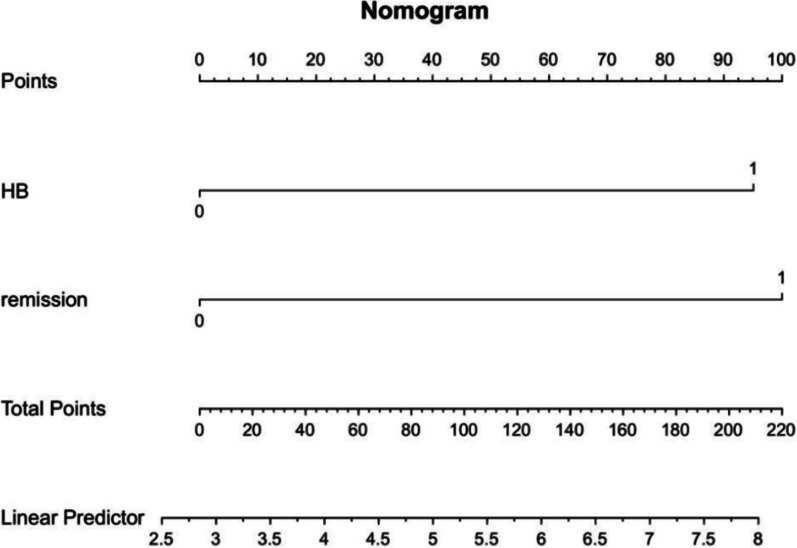
The nomogram of risk factors

According to the data we observed in the multivariate analysis, as presented in Table 3 relief of bowel obstruction symptoms after NITs insertion (OR = 0.123, 95% CI 0.030, 0.504, *p* = 0.004) and hemoglobin before NITs insertion (OR = 0.958, 95% CI 0.926, 0.991, *p* = 0.014) had excellent performance in prediction of SAEs (Fig. 4). In addition, the composite index, called ‘risk score system’, had the highest AUC (0.840, 95%CI 0.727, 0.953) for predicting the occurrence of SAEs (Tables 4 and 5). A nomogram containing these two factors has been built up to predict the occurrence of SAEs, which presents increasing rates of SAEs with accumulation of risk scores (Fig. 5).

**Table 4:** The predictive effectiveness of new indicators-score

Score	The occurrence of SAEs	*p*
0	(1/74)1.3%	< 0.001
1	(6/51)11.7%	
2	(6/11)54.5%	

All *p* values < 0.05 were considered statistically significant

**Table 5:** ROC curve

	AUC	95%CI	*p*
adHB	0.789	(0.685,0.893)	0.001
Remission	0.737	(0.575,0.900)	0.005
Score	0.840	(0.727,0.953)	< 0.001

adHB, hemoglobin before NITs insertion; Remission, abdominal pain or distension relieved after NITs insertion

All *p* values < 0.05 were considered statistically significant

### A special case in succeed group

**Fig. 6:**
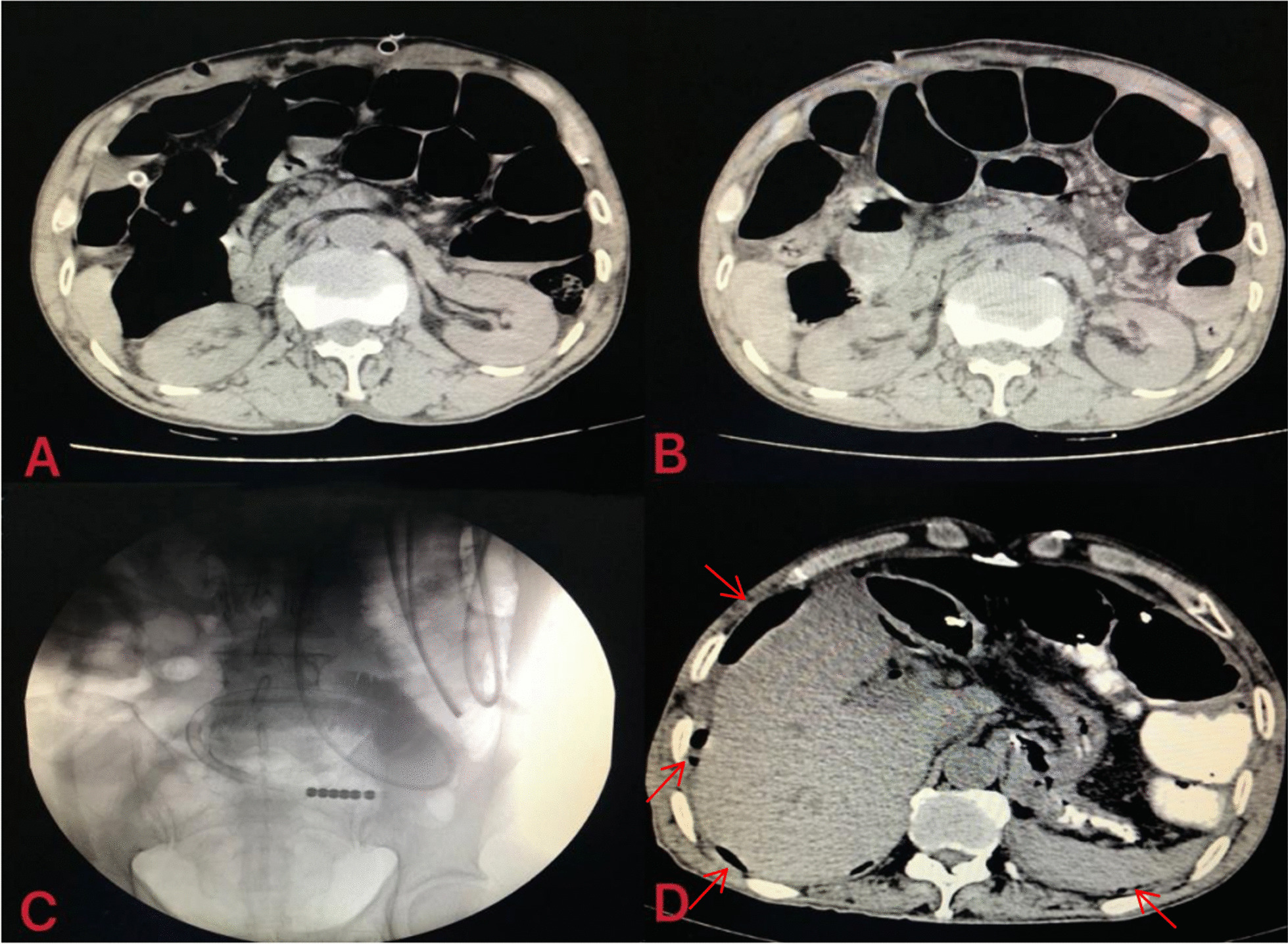
CT image of patient with SAE in the success group. **A** the first day postoperation, bowel expansion; **B** the day before tubes placement, after thirteen days medicine treatment, the bowel is still dilated; **C** tubes insertion; **D** two days after tube insertion(at the arrow is subphrenic free air)

It is important to note that one case in the success group experienced gastrointestinal perforation. The patient accepted left liver resection and diagnosed as intestinal obstruction on the 13th day postoperatively (Fig. 6A, B), then underwent NIT insertion followingly (Fig. 6C). Unfortunately, gastrointestinal perforation and abdominal infection occurred (Fig. 6D). After continuous abdominal irrigation, antibiotic therapy and infusion of plasma, the patient was able to eat semiliquid diet and discharged on the 30th day postoperatively. This case illustrated that even if SAEs occurred after NIT placement, effective decompression through the tube can lead to successful conservative treatment and avoid reoperation.

## Discussion

SBO is a common cause of emergency department visits, and there is a dilemma in the management of SBO: conservative treatment may result in delayed intestinal ischemia and necrosis; However, unnecessary emergency surgery could increase the incidence of severe complications and the formation of new adhesive bands. With the development of instruments, the usage of NITs could lead to a reduction of intestinal pressure in the early-stage and avoid intestinal ischemia, thus transforming the case from acute SBO to subacute SBO.

Previous studies have confirmed the efficacy of nasointestinal decompression through NITs for SBO [6, 12–15]; Similarly, in our center, NITs have confirmed therapeutic efficacy for SBO. The patients who have success with NITs decompression could avoid electrolyte imbalance and postoperative pneumonia, with a shortened length of hospital-stay and decreased hospital costs. However, in patients with tumors, the success rate of NITs insertion was significantly lower, which may owe to peritoneal metastasis that triggered mechanical intestinal obstruction in several portions, cannot be alleviated by single-one obstructive site decompression. Meanwhile, tumors accounted for 61.5% of the causes of obstruction in the SAE group and 19.5% in the non-SAE group (*p* = 0.002). These results highlight that patients with malignant bowel obstruction could barely benefit form NITs insertion. HAN’s study showed that the cure rate of NITs for benign ileus was significantly higher than that for malignant ileus (38.1% vs. 6.7%, respectively; *p* = 0.01) [17]. As a result of a poor nutritional status, systemic deterioration and the multiple portions obstructions found in malignant small bowel obstruction, hampered the strategies for the treatment. Only 30–48.9% of the patients can be successfully treated, as mentioned in the literature. Patients with malignant bowel obstruction should be carefully selected for treatment with the aim of improving their quality of life [21–23].

Our research found that hypochloremia in the success group was lower than that in the failed group, which was related to the findings that hypochloremia closely increased mortality rates in severe patients [24–26]. We speculated that hypochloremia could be a marker of malnutrition in the patient before surgery. Moreover, it could be related to the status of preoperative chronic kidney disease and known heart failure with hypochloremia [27, 28]. In addition, insufficient fluid supplementation before surgery and fluid accumulation in the third space may lead to prerenal renal insufficiency, and during decompression, the electrolyte support cannot meet the body's needs, which may lead to the occurrence of hypochloremia after NITs insertion.

Even if the patient failed to achieve the goal of complete alleviation of SBO, NITs drainage of the luminal contents could reduce the internal pressure, ease bowel edema and find the cause of the obstruction. Moreover, the incidence of SAE was extremely low in all patients with NITs insertion (9.6%) (success group vs failure group: 1.5% vs 17.6%) (Table 6) [16, 29, 30]. We compared some previous studies on the incidence of complications during conservative treatment of intestinal obstruction [2, 31, 32]. It was obvious that the incidence of SAEs after NITs insertion in our study was not significantly higher than other conservative treatment (Table 7 and 8). Different from previous study, no patients suffered with intestinal ischemia in our study, however, Intractable diarrhea and Methicillin-resistant staphylococcus aureus colitis occurred. Consistent with the report by Shogo Tanaka, patients after NITs insertion should pay attention to their intestinal flora and avoid the use of antibiotics (Table 9).

**Table 6:** Previous studies on complications after NITs insertion

	Sample	Complication case	Complication
Fleshner PR	27	3 (11.1%)	Pneumonia, Colo cutaneous fistula
Takumi Sakakibara	91	8 (8.8%)	NR
Shogo Tanaka	53	5 (9.5%)	Intractable diarrhea, Wound infection, Methicillin-resistant staphylococcus aureus colitis, Renal failure, Leakage from anastomosis
This article	136	13 (9.6%)	Multiple reasons, Give up treatment, Respiratory failure, Gastrointestinal perforation, Severe pneumonia, Anastomotic fistula, Septic shock, Severe metabolic acidosis

*NR* not reported

**Table 7:** Baseline characteristics of included studies

Study	Country	Journal	Design	Follow up	presentation
Millet [2]	France	Radiology	Retrospective cohort	1 month	Nonsurgical treatment in patients with ASBO
Khalil [33]	Egypt	The Egyptian Journal of Surgery	RCT	2 years	Early laparoscopic adhesiolysis versus conservative treatment of recurrent ASBO
Fevang [31]	Norway	Eur J Surg	Prospective cohort	NR	To evaluate the outcome after initial non-operative treatment in patients with SBO
Miller [32]	Canada	British journal of surgery	Retrospective cohort	6 years	To note the long-term prognosis and recurrence rates in ASBO for operative and non-operative treatment

*NR* not reported

**Table 8:** Baseline characteristics of included population

Study	Sample size	Age	Male	Diagnosis basis	Complication	Rate (%)
Millet [2]	159	69	95 (59.7%)	Clinical and radiological	Intestinal ischemia	10
Khalil [33]	25	56.1	12 (48.0%)	Clinical and radiological	Pneumonia, Nostril erosion	16
Fevang [31]	93	NR	NR	Clinical and radiological	Pneumonia, Cardiac complications, Thrombosis or embolism, Respiratory insufficiency,	8.4
Miller [32]	267	NR	NR	Clinical and radiological	later episode of strangulate bowel	2.9

*NR* not reported

**Table 9:** Comparison of complications between conservative treatment and NIT treatment

	Conservative treatment	NIT treatment
Intestinal ischemia	√	×
Pneumonia	√	√
Respiratory failure	√	√
Cardiac complications	√	×
Thrombosis or embolism	√	×
Nostril erosion	√	×
Gastrointestinal perforation	×	√
Severe metabolic acidosis	×	√
Intractable diarrhea	×	√
Methicillin-resistant staphylococcus aureus colitis	×	√
Renal failure	×	√

We also constructed a risk score system containing the relief of bowel obstruction symptoms and preoperative anemia, to predict the occurrence of SAEs after NITs insertion. Failure in decompression will lead to respiratory complications, while successful relief of bowel obstruction symptoms by NITs insertion can reduce the intra-abdominal pressure and the abdominal pain of the patients, thus improving respiratory ventilation and effectively reduce the incidence of pneumonia. Similarly, preoperative anemia also contributed to an increased risk of the occurrence of SAEs. As a carrier of oxygen, a reduction of hemoglobin may indicate intestinal ischemia and an insufficient oxygen support. Therefore, preoperative anemia should be corrected before NITs insertion to minimize the risk.

The present study is a retrospective cohort study that focuses on SAEs after NITs insertion using the PSM scoring system to detect the indication for NITs insertion. We constructed a risk score system including the hemoglobin level before NITs insertion and the relief of bowel obstruction symptoms after NITs insertion, to predict the occurrence of SAEs. There were some limitations in this study. Firstly, this was a retrospective study in a single center; thus, we will initiate a prospective, multicenter study to confirm our findings. Secondly, in this paper, data on conservative treatment of patients with SBO were lacking, and the complication rate of conservative treatment could only be determined by referring to previous studies. Thirdly, the severity of the intestinal obstruction in patients with conservative treatment may be generally mild, so NITs treatment or surgical treatment is not performed. It is better to further evaluate the degree of obstruction in combination with CT imaging data to evaluate the incidence of SAEs (Additional files 1 and 2).

## Conclusion

NITs successfully insertion could avoid SAEs occurrence in SBO conservative treatment. SBO patients without anemia and could be relieved after NITs insertion could be the potential benefit group for this therapy.

## Supplementary Information

**Additional file 1.** Supplementary Figure.

**Additional file 2.** Supplementary Table.

## Data Availability

The datasets generated and/or analysed during the current study are not publicly available due to information safety but are available from the corresponding author on reasonable request.

## Abbreviations

NIT: Nasointestinal tube
SBO: Small bowel obstruction
SAE: Severe adverse events
AUC: Area under curve
ROC: Receiver operating characteristic
CD: Clavien–Dindo grading of surgical complication
NLR: Neutrophils/lymphocytes
WBC: White blood cells
RBC: Red blood cell
Ne: Neutrophils
HB: Hemoglobin
APTT: Activated partial thromboplastin time
PLT: Blood platelet
