# Supplementary figures and images for: Who would avoid severe adverse events from nasointestinal tube in small bowel obstruction? A matched case–control study
Source: BMC Gastroenterol. 2022 Jul 7;22:332. doi: 10.1186/s12876-022-02405-8 (PMC9264659; doi:10.1186/s12876-022-02405-8)

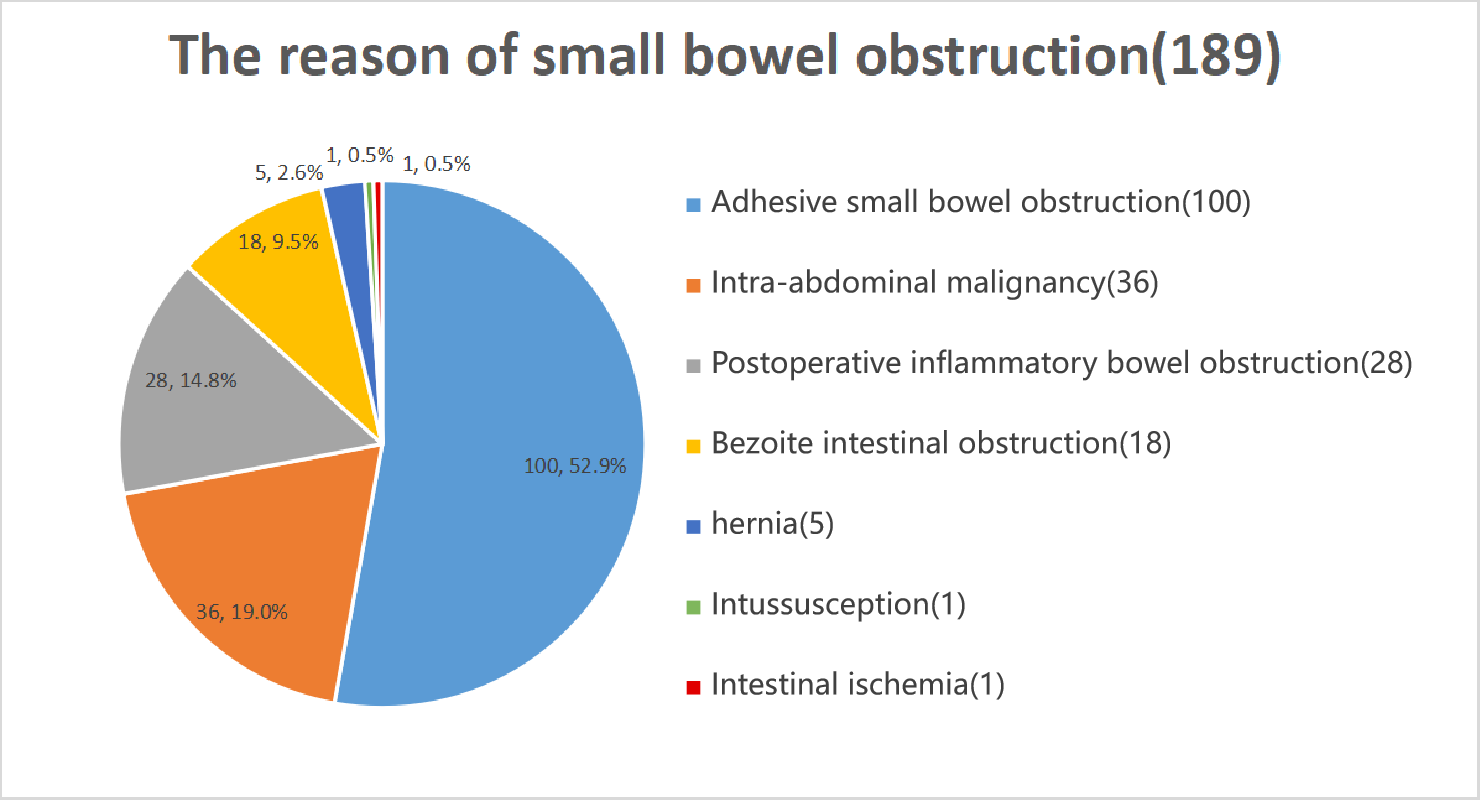

Supplement: Supplementary file 1 — Additional file 1. Supplementary Figure. [file 12876_2022_2405_MOESM1_ESM.png]
