# Supplementary material for: Who would avoid severe adverse events from nasointestinal tube in small bowel obstruction? A matched case–control study
Source: BMC Gastroenterol. 2022 Jul 7;22:332. doi: 10.1186/s12876-022-02405-8 (PMC9264659; doi:10.1186/s12876-022-02405-8)
Supplement: Supplementary file 2 — Additional file 2. Supplementary Table. [file 12876_2022_2405_MOESM2_ESM.docx]

**Supplementary Table 1:The Clinical characteristics of SAEs**

|  | Overall  (n=136) | Gender | Age | Obstruction  reason | Remission | Fees  (¥) | Length  (days) |
| --- | --- | --- | --- | --- | --- | --- | --- |
| Multiple reasons | 4(2.9%) | male | 38 | tumor | Yes | 183121 | 57 |
| Multiple reasons |  | male | 56 | tumor | none | 26033 | 5 |
| Multiple reasons |  | female | 71 | tumor | none | 839381 | 14 |
| Multiple reasons |  | male | 70 | adhesive | Yes | 39849 | 8 |
| Treatment at another hospital | 2(1.4%) | female | 49 | adhesive | Yes | 136611 | 37 |
| Treatment at another hospital |  | male | 36 | tumor | none | 96289 | 43 |
| Respiratory failure | 2(1.4%) | male | 67 | adhesive | none | 369100 | 63 |
| Respiratory failure |  | female | 77 | tumor and adhesive | Yes | 73627 | 18 |
| Gastrointestinal perforation | 1（0.7%） | male | 66 | inflammatory | Yes | 124824 | 40 |
| Severe pneumonia | 1（0.7%） | male | 68 | tumor | none | 96701 | 19 |
| Anastomotic fistula | 1（0.7%） | male | 62 | tumor | none | 89155 | 16 |
| Septic shock | 1（0.7%） | male | 64 | adhesive | none | 140474 | 50 |
| Severe metabolic acidosis | 1（0.7%） | female | 37 | tumor | none | 127480 | 37 |

|  | Cl^-^ level | adRBC | adHB | adAPTT | HB | WBC | Pulmonary Infection |
| --- | --- | --- | --- | --- | --- | --- | --- |
| Multiple reasons | 0 | 2.92 | 93 | 51.5 | 104 | 7.50 | none |
| Multiple reasons | 3 | 2.20 | 63 | 47.6 | 49 | 40.00 | none |
| Multiple reasons | 3 | 3.18 | 85 | 92.3 | 82 | 7.26 | none |
| Multiple reasons | 1 | 4.44 | 115 | 36.6 | 93 | 14.56 | none |
| Treatment at another hospital | 1 | 3.08 | 91 | / | 97 | 8.90 | none |
| Treatment at another hospital | 3 | 3.19 | 89 | 37.7 | 101 | 9.42 | none |
| Respiratory failure | 1 | 3.84 | 108 | 38.2 | 108 | 11.41 | none |
| Respiratory failure | 1 | 2.64 | 83 | 38.7 | 87 | 5.20 | none |
| Gastrointestinal perforation | 3 | 3.28 | 107 | 42.9 | 108 | 11.85 | none |
| Severe pneumonia | 1 | 3.36 | 100 | 45.4 | 83 | 10.82 | yes |
| Anastomotic fistula | 0 | 4.86 | 127 | 47.4 | 115 | 10.15 | none |
| Septic shock | 3 | 3.96 | 116 | 35.7 | 130 | 14.31 | yes |
| Severe metabolic acidosis | 1 | 3.61 | 100 | 37.9 | 101 | 8.40 | none |

**(continued table)**

SAE: severe adverse event; Remission: abdominal pain or distension relieved after NITs insertion; Cl^-^ level: level of chloride ion; adRBC: red blood cell before NITs insertion; adHB: hemoglobin before NITs insertion; adAPTT: activated partial thromboplastin time before NITs insertion; HB: hemoglobin after NITs insertion; WBC: white blood cell after NITs insertion.
